# Correction: Factors associated with change in adherence to COVID-19 personal protection measures in the Metropolitan Region, Chile

**DOI:** 10.1371/journal.pone.0320960

**Published:** 2025-03-21

**Authors:** Simón Varas, Felipe Elorrieta, Claudio Vargas, Pablo Villalobos Dintrans, Claudio Castillo, Yerko Martinez, Andrés Ayala, Matilde Maddaleno

The Funding statement for this paper is incorrect. The correct Funding statement is as follows: The authors gratefully acknowledge financial support from the grant DICYT 022191MH_DAS “Understanding causes and consequences of COVID-19 and other diseases in public health: multicausality, temporality and spatiality applied to decision making”, Universidad de Santiago de Chile.

## References

[1] Varas S, Elorrieta F, Vargas C, Villalobos Dintrans P, Castillo C, Martinez Y, et al. Factors associated with change in adherence to COVID-19 personal protection measures in the Metropolitan Region, Chile. PLoS One. 2022;17(5): e0267413. doi: 10.1371/journal.pone.0267413 35551277 PMC9098054

