# Supplementary material for: Factors associated with change in adherence to COVID-19 personal protection measures in the Metropolitan Region, Chile
Source: PLoS One. 2022 May 12;17(5):e0267413. doi: 10.1371/journal.pone.0267413 (PMC9098054; doi:10.1371/journal.pone.0267413)
Supplement: S1 File — (DOCX) [file pone.0267413.s002.docx]

**Supplementary material:** Questionnaire of adherence to self-care measures to COVID-19 personal protection measures in the Metropolitan Region, Chile.

The Universidad de Santiago de Chile (USACH), through the Vice-Rectory for Research and Development, is conducting a study on the behavior of people in relation to recommended practices in the context of the SARS-CoV-2 pandemic, also known as COVID-19 or Coronavirus.

We invite you to answer this questionnaire. It will not take more than 10 minutes. To answer this survey you must be over 18 years of age and reside in the Metropolitan Region.

We remind you that there are no correct or wrong answers and that all the information provided will only be used for the purposes of this study. We assure you total anonymity. In addition, all the information is protected by the statistical secrecy set in Law N ° 17.374, to which the USACH adheres.

Thank you very much for your participation.

1. Name __________________

2. Sex:

1. Male
2. Female
3. non-binary
4. Trans
5. I would rather not say
6. Other

4. Age:________

5. Average Monthly Household Income in the last three months:

1. Less than CL$500.000
2. Between CL$500.000 and CL$1.500.000
3. More than CL$1.500.000

6. Area of residence

1. Alhué
2. Buin
3. Calera de Tango
4. Cerrillos
5. Cerro
6. Navia
7. Colina
8. Conchalí
9. Curacaví
10. El Bosque
11. El Monte
12. Estación Central
13. Huechuraba
14. Independencia
15. Isla de Maipo
16. La Cisterna
17. La Florida
18. La Granja
19. La Pintana
20. La Reina
21. Lampa
22. Las Condes
23. Lo Barnechea
24. Lo Espejo
25. Lo Prado
26. Macul
27. Maipú
28. María Pinto
29. Melipilla
30. Ñuñoa
31. Padre Hurtado
32. Paine
33. Pedro Aguirre Cerda
34. Peñaflor
35. Peñalolén
36. Pirque
37. Providencia
38. Pudahuel
39. Puente Alto
40. Quilicura
41. Quinta Normal
42. Recoleta
43. Renca
44. San Bernardo
45. San Joaquín
46. San José de Maipo
47. San Miguel
48. San Pedro
49. San Ramón
50. Santiago
51. Talagante
52. Til Til
53. Vitacura

7. Phone number:____________

8. e-mail:_____________

9. Pollster’s name:______________

COVID-19 questions

10. Regarding facemask use, which of the following sentences fits your behavior the best? TODAY

1. I ALWAYS use it, correctly and even if nobody obliges me to do so.
2. I use it MOST OF THE TIMES, although not always correctly.
3. I use it ONLY SOMETIMES, in general when it is mandatory and not always correctly.
4. I ALMOST NEVER wear a facemask and I only wear it when I am forced to do so.
5. Don´t know/ don`t answer

11. Regarding facemask use, which of the following sentences fits your behavior the best? DURING THE YEAR 2020

1. I ALWAYS use it, correctly and even if nobody obliges me to do so.
2. I use it MOST OF THE TIMES, although not always correctly.
3. I use it ONLY SOMETIMES, in general when it is mandatory and not always correctly.
4. I ALMOST NEVER wear a facemask and I only wear it when I am forced to do so.
5. Don´t know/ don`t answer

12. Regarding handwashing and/ or the use of alcohol gel, which of the following sentences fits your behavior the best? TODAY

1. When I get somewhere, I ALWAYS wash my hands with soap and water for at least 20 seconds or use alcohol gel.
2. When I get somewhere, I USUALLY wash my hands with soap and water for a few seconds or use alcohol gel.
3. When I get somewhere, I ONLY SOMETIMES wash my hands with soap and water for a few seconds or use alcohol gel.
4. I ALMOST NEVER wash my hands or use alcohol gel when I get somewhere, unless I am obliged to do so.
5. Don´t know/ don`t answer

13. Regarding handwashing and/ or the use of alcohol gel, which of the following sentences fits your behavior the best? DURING THE YEAR 2020

1. When I get somewhere, I ALWAYS wash my hands with soap and water for at least 20 seconds or use alcohol gel.
2. When I get somewhere, I USUALLY wash my hands with soap and water for a few seconds or use alcohol gel.
3. When I get somewhere, I ONLY SOMETIMES wash my hands with soap and water for a few seconds or use alcohol gel.
4. I ALMOST NEVER wash my hands or use alcohol gel when I get somewhere, unless I am obliged to do so.
5. Don´t know/ don`t answer

14. Regarding Social Distancing measures, which of the following sentences fits your behavior the best?: TODAY

1. I ALWAYS keep physical distance and do not attend events with family, friends or acquaintances.
2. I USUALLY keep physical distance and rarely attend events with family, friends, or acquaintances.
3. FEW TIMES I keep my physical distance and I usually attend events with family, friends or acquaintances.
4. I ALMOST NEVER keep physical distance and I regularly attend events with family, friends or acquaintances.
5. Don´t know/ don`t answer

15. Regarding Social Distancing measures, which of the following sentences fits your behavior the best?: DURING THE YEAR 2020

1. I ALWAYS keep physical distance and do not attend events with family, friends or acquaintances.
2. I USUALLY keep physical distance and rarely attend events with family, friends, or acquaintances.
3. FEW TIMES I keep my physical distance and I usually attend events with family, friends or acquaintances.
4. I ALMOST NEVER keep physical distance and I regularly attend events with family, friends or acquaintances.
5. Don´t know/ don`t answer

16. Are you vaccinated?

1. Yes, I am vaccinated (at least with one dose)
2. No, but I hope to.
3. No, and I haven't decided yet if I'm going to do it.
4. No, I don't want to get vaccinated.
5. Don´t know/ don`t answer

17. Comments or Observations:
